# Effects of short‐term exercise on plasma metabolic and lipidomic profiles of individuals with type 2 diabetes

**DOI:** 10.1113/EP092768

**Published:** 2025-06-23

**Authors:** Maartje Cox, Aaron Raman, Timothy Fairchild, John P. Beilby, Bu B. Yeap, Jeremy K. Nicholson, Julien Wist, Jeremiah Peiffer, Nathan G. Lawler

**Affiliations:** ^1^ Australian National Phenome Centre, Health Futures Institute, Harry Perkins Institute of Medical Research Murdoch University Perth Western Australia Australia; ^2^ School of Allied Health, College of Health and Education Murdoch University Perth Western Australia Australia; ^3^ Centre for Healthy Ageing, Health Futures Institute Murdoch University Perth Western Australia Australia; ^4^ School of Biomedical Sciences University of Western Australia Perth Western Australia Australia; ^5^ Medical School University of Western Australia Perth Western Australia Australia; ^6^ Department of Endocrinology and Diabetes Fiona Stanley Hospital Perth Western Australia Australia; ^7^ Centre for Computational and Systems Medicine, Health Futures Institute, Harry Perkins Institute Murdoch University Perth Western Australia Australia; ^8^ Faculty of Medicine, Department of Metabolism, Digestion and Reproduction, Division of Digestive Diseases Imperial College London UK; ^9^ Chemistry Department Universidad del Valle Cali Colombia

**Keywords:** exercise, lipidomics, metabolomics, oral glucose tolerance test, type 2 diabetes mellitus

## Abstract

Type 2 diabetes mellitus (T2DM) is a common metabolic disorder characterized by chronic hyperglycaemia, with physical inactivity and excessive adiposity as predisposing factors. This clinical trial aimed to investigate the effects of an exercise intervention on the metabolome of T2DM participants, fasting and in response to an oral glucose tolerance test (OGTT) and an acute exercise stimulus. Thirteen people with T2DM (age 51 ± 7 years; body mass index 32.7 ± 4.9 kg/m^2^) completed 45 min of moderate‐intensity treadmill exercise on 12 days consecutively. Blood samples were collected before and after the first and last training sessions and during a pre‐ and postintervention OGTT. Fasted blood samples were collected from 198 healthy control subjects and 208 people with T2DM from an independent cohort for comparison. Samples were analysed using high‐resolution ^1^H nuclear magnetic resonance spectroscopy and liquid chromatography–mass spectrometry. The exercise intervention did not induce a shift towards a healthier fasted metabolome in people living with T2DM. In response to consumption of a glucose bolus (OGTT), glycolysis‐related metabolites increased and free fatty acids decreased, with no effect of the exercise intervention. In response to acute exercise, glucose and amino acids decreased and free fatty acids increased, with similar responses on the last day of training as on the first day, indicating no effect of the intervention. The clinical trial was registered prospectively in the Australian New Zealand Clinical Trials Registry ACTRN12617000286347 on 24 February 2017.

## Abstract (highlights)

- **What is the central question of this study?**
  People with type 2 diabetes mellitus (T2DM) have a different metabolic phenotype and an impaired response to an oral glucose tolerance test compared with healthy individuals. It is unknown whether a short‐term exercise programme can improve this response.
- **What is the main finding and its importance?**
  Twelve consecutive days of moderate‐intensity exercise do not improve the fasted metabolic phenotype or oral glucose tolerance test response in people with T2DM. These findings suggest that exercise‐induced metabolic benefits in T2DM might occur only after longer‐term engagement in exercise activities.

## 1 INTRODUCTION

Type 2 diabetes mellitus (T2DM) is a highly prevalent (>400 million patients worldwide) metabolic disorder characterized by chronically elevated blood glucose resulting from systemic insulin resistance and impaired insulin secretion (WHO, 2023; Zheng et al., 2018). Individuals with T2DM are at increased risk of developing microvascular complications, including neuropathy, retinopathy and nephropathy, in addition to cardiovascular diseases leading to premature mortality (Zheng et al., 2018).

People living with T2DM have chronically elevated (≥7.0 mmol/L) fasting blood glucose concentrations and demonstrate exaggerated blood glucose responses to ingestion of glucose. The oral glucose tolerance test (OGTT) is, therefore, a useful diagnostic tool, because it enables the investigation of both fasted and postprandial glucose disturbances in individuals at risk of T2DM and those with T2DM (Gao et al., 2022; Wang et al., 2019). In response to the ingestion of a standardized (typically 75 g) glucose solution, the body shifts from a fasted to a fed state, leading to abrupt changes in metabolic processes to restore glucose homeostasis. Lowering the fasting and postprandial glucose concentrations is key to improving glycaemic control for T2DM, and exercise has been shown to achieve this (Colberg et al., 2010; Kanaley et al., 2022; Kirwan et al., 2017; Syeda et al., 2023). Not only does exercise improve glycaemic control, it also induces improvements in insulin sensitivity, blood lipid profile and inflammation in individuals with T2DM (American Diabetes Association, 2018; Colberg et al., 2010, 2016; Kadoglou et al., 2007; Kanaley et al., 2022; Kirwan et al., 2017, 2009; Magkos et al., 2020; Pan et al., 2018; Syeda et al., 2023). Although the combination of aerobic and resistance training appears most efficacious (reviewed by Schwingshackl et al., 2014), consistent aerobic training alone has been shown to improve glycaemic control (reviewed and meta‐analysed by Snowling & Hopkins, 2006). Although life‐long exercise is advocated, it has been shown that six cycling sessions over a 2 week period (Little et al., 2011) and 7 days of daily aerobic cycling and/or walking exercise can improve insulin sensitivity and glucose concentrations in otherwise sedentary individuals with T2DM (Kirwan et al., 2009; Mikus et al., 2011, 2012; O'Gorman et al., 2006). Importantly, it has been proposed that exercise has the capacity to achieve glycaemic outcomes similar to conventional treatment, such as pharmacological therapy (Boulé et al., 2001; Snowling & Hopkins, 2006).

The beneficial effects of exercise training on glycaemic control and insulin sensitivity are well established; however, the additional metabolic processes associated with and potentially enabling these beneficial changes are yet to be elucidated. For instance, multiple alternative pathways, such as the hexosamine, polyol, pentose phosphate and the glyoxalase pathway are associated with the metabolism of exogenous glucose and might be upregulated in T2DM (Gallagher et al., 2016; Liu et al., 2013; Yan, 2018). Metabolomics investigates metabolites, which are the substrates and products of metabolism driving essential cellular functionalities (Johnson et al., 2016), in biological specimens. As such, metabolic phenotyping offers the possibility to examine the effects of exercise on polar metabolites, including amino acids, sugars, lipoproteins and fatty acids (Chen et al., 2022), and non‐polar metabolites, such as lipids. Lipidomics strives to map the cellular/tissue lipidome by quantifying multiple lipid species (Wang et al., 2020). Broadening molecular phenotypes by combining all accessible molecules provides a thorough overview of the metabolic landscape (Belhaj et al., 2021) and allows for the identification of critical metabolic drivers in the pathology of T2DM (Wang et al., 2020), in addition to the subsequent changes in metabolites during remission from T2DM. Both NMR spectroscopy and mass spectrometry have proved highly effective in studying the systemic effects of diabetes for >40 years (Bales et al., 1984; Liebich, 1983), and here we took a combined approach in this study.

Previous research has sought to identify metabolic signatures associated with T2DM, with differences in metabolites such as (branched‐chain) amino acids (Ahola‐Olli et al., 2019; Floegel et al., 2013; Gu et al., 2020; Liu et al., 2017; Lu et al., 2016; Merino et al., 2018; Mitro et al., 2021; Palmer et al., 2015; Peddinti et al., 2017; Qiu et al., 2016; Rebholz et al., 2018; Shi et al., 2018; Stančáková et al., 2012; Tillin et al., 2015; Vangipurapu et al., 2019; Wang et al., 2011; Wang et al., 2019; Wang‐Sattler et al., 2012; Yang et al., 2018), triglycerides (Ahola‐Olli et al., 2019; Chen et al., 2020; Festa et al., 2005; Lee et al., 2016; Liu et al., 2017; Mahendran et al., 2013; Mamtani et al., 2016; Mora et al., 2010; Prada et al., 2021; Suvitaival et al., 2018; Wang et al., 2019), cholesterol (Ahola‐Olli et al., 2019; Chen et al., 2020; Festa et al., 2005; Fizelova et al., 2015; Liu et al., 2017; Mora et al., 2010; Sokooti et al., 2021), phospholipids (Festa et al., 2005; Lee et al., 2016; Mamtani et al., 2016; Mora et al., 2010; Prada et al., 2021; Suvitaival et al., 2018), ceramide (Lee et al., 2016; Mamtani et al., 2016; Prada et al., 2021; Suvitaival et al., 2018), diacylglycerols (Shi et al., 2018), free fatty acids (FFAs) (Ouyang et al., 2021; Sobczak et al., 2019), phosphatidylcholine (Lee et al., 2016; Mamtani et al., 2016; Prada et al., 2021; Suvitaival et al., 2018), lysophosphatidylcholine (Fall et al., 2016; Floegel et al., 2013; Lee et al., 2016; Mamtani et al., 2016; Ouyang et al., 2021; Prada et al., 2021; Shi et al., 2018; Suvitaival et al., 2018; Wang‐Sattler et al., 2012) and lysophosphatidylethanolamines (Lee et al., 2016; Mamtani et al., 2016; Prada et al., 2021; Suvitaival et al., 2018). Furthermore, Wang et al. (2019) studied the metabolite response to an OGTT in individuals with insulin resistance but normal glucose tolerance in comparison to insulin‐sensitive individuals with normal glucose tolerance. They found greater increases in glucose at 2 h postprandial but smaller increases in alanine, lactate, pyruvate, branched‐chain amino acids (BCAAs), ketone bodies and triglycerides in those with insulin resistance in comparison to the insulin‐sensitive individuals (Wang et al., 2019).

More recently, the effects of a short‐term exercise intervention (six low‐volume, high‐intensity interval training sessions over 2 weeks) on the plasma metabolome were investigated in individuals with T2DM (Savikj et al., 2022). Their study found that lipids (i.e., fatty acids, diacylglycerols, acyl‐carnitines and primary bile acids) were increased in fasting blood plasma following the exercise intervention (Savikj et al., 2022). Increases in lipid mobilization and mitochondrial substrate shuttling following the high‐intensity interval training exercise intervention were postulated to underlie these findings (Savikj et al., 2022). Whether the acute metabolic response to exercise is altered following a period of exercise training and how the postprandial metabolite response is altered with exercise training in individuals with T2DM remain to be established. Additionally, it remains unclear whether metabolic markers associated with T2DM shift closer towards a healthy state or remain at levels typical for T2DM following exercise training. This study aims to address these gaps by investigating whether a 12 day exercise intervention changes the metabolic response to an acute exercise bout or to an OGTT. It also compares a targeted panel of T2DM‐related metabolites in the fasted plasma of individuals with T2DM, pre‐ and postintervention, with those of healthy control subjects and T2DM control subjects from an independent cohort. It was hypothesized that the glucose response would improve with exercise and that the metabolic fingerprint of people with T2DM would shift closer towards a healthy fingerprint.

## 2 MATERIALS AND METHODS

### 2.1 Ethical approval

The exercise intervention and Busselton Healthy Ageing Study (BHAS) were conducted in accordance with the *Declaration of Helsinki* and comply with the journal's human ethics policy. All included participants provided written informed consent. The study protocol was approved by the Human Research Ethics Committee of Murdoch University (2015/220) and was registered prospectively as a clinical trial with the Australian New Zealand Clinical Trials Registry (ANZCTR) under ACTRN12617000286347. The primary outcomes of this clinical trial were glucose tolerance and regulatory T‐cell response to a short‐term exercise intervention in individuals with T2DM. The present study reports findings from the metabolomics branch of the trial, which was subsequently added as a secondary outcome.

### 2.2 Participants

Participants were eligible for inclusion if they had a previous diagnosis of T2DM, a body mass index (BMI) ≥ 25 kg/m^2^, aged between 18 and 65 years and performed less than three bouts of low‐ to moderate‐intensity exercise per week. Participants were excluded if they used exogenous insulin, smoked, or if there were contraindications to exercise as determined by the Exercise & Sport Science Australia (ESSA) medical screening tool. Data collection occurred between March 2017 and December 2017.

To identify meaningful intervention‐induced changes in the metabolome of our T2DM participants, we included a healthy control group and a T2DM control group (T2DM controls) from an independent cohort. Blood samples from healthy controls and T2DM controls were selected from participants of the first and second wave of the BHAS. The BHAS is a population‐based study, in which adults born between 1946 and 1964 living in the City of Busselton, Western Australia, took part. BHAS participants defined as ‘very healthy’ based on previously published criteria, and with BMI ≤ 25 kg/m^2^, were included in the healthy control group of the present study (Masuda et al., 2023). BHAS participants with confirmed T2DM and whose BMI matched the BMI of T2DM participants taking part in the exercise intervention were included in the T2DM‐controls group. Fasted blood samples collected from these BHAS participants were used for the present study and processed in the same way as samples from people with T2DM undergoing the exercise intervention using nuclear magnetic resonance (NMR) spectroscopy methods (described in section 2.4.2). Samples from BHAS participants were collected between 2010 and 2015 (wave 1) and between 2016 and 2021 (wave 2).

### 2.3 Study design for exercise intervention

**FIGURE 1:**
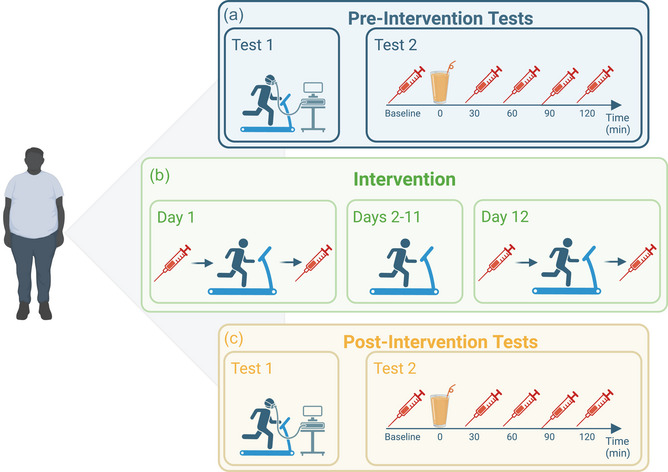
Schematic diagram of the study design. (a, c) The pre‐intervention tests (a) and postintervention tests (c) were identical and consisted of an incremental exercise test on a treadmill and an OGTT. (b) The intervention comprised 12 days of structured exercise, where blood samples were collected before and after the first and last training sessions (Figure 1b). Illustration created with Biorender.com. Abbreviation: OGTT, oral glucose tolerance test.

This single‐arm clinical trial consisted of two pre‐intervention visits, a 12 day intervention period, and two postintervention visits. An overview of the study design is provided in Figure 1, and a detailed description is given below. All measurements and training sessions were performed at Murdoch University's Exercise Physiology Laboratory. One minor deviation from the original trial protocol is the minimum BMI of T2DM participants, which was reduced to BMI ≥ 25 kg/m^2^ to ensure that no potential overweight participants with T2DM were excluded.

#### 2.3.1 Pre‐intervention visits

During the first pre‐intervention session, anthropometrical parameters, including age, height, weight and BMI, were determined. Additionally, dual X‐ray absorptiometry (DXA) scanning (Hologic Discovery QDR series, Hologic Inc., Bedford, MA, USA) was performed for the determination of fat percentage and visceral adipose tissue (VAT). Subsequently, participants completed an incremental exercise test on a treadmill (Trackmaster, Full Vision, KS, USA) according to the following protocol: starting velocity was 2.5 km/h and was increased by 1 km/h every 2 min until a velocity of 5.5 km/h was reached. Then, a 2% increase in gradient was applied until volitional exhaustion. Ventilation was measured continuously throughout the exercise test as 15 s averages by a metabolic cart (Parvomedics TrueOne, Sandy, UT, USA), and peak oxygen uptake (${\dot{V}}_{O_{2}\mathrm{peak}}$) was defined as the highest 30 s average oxygen uptake (${\dot{V}}_{O_{2}}$) measured during the last stage of the incremental test. Heart rate was measured at a rate of 1 Hz (Polar T31, Kempele, Finland).

At least 2 days after the ${\dot{V}}_{O_{2}\mathrm{peak}}$ test, participants reported to the laboratory in a fasted state for their second pre‐intervention visit, which involved a first OGTT (OGTT 1). Participants were instructed to refrain from exercise and medication intake on the morning of the OGTT, and to refrain from nutriments with high sugar content in the evening preceding the OGTT. Participants were asked to record their food intake on the day before the OGTT. Venous blood samples (8 mL) were taken from the antecubital vein prior to consumption of a drink containing 75 g of glucose and at 30 min intervals following consumption, up to and including 120 min postconsumption. Thus, five blood samples were collected in total. The time points (TPs) of blood sample collection during an OGTT are hereafter referred to as tp_0_, tp_30_, tp_60_, tp_90_ and tp_120_, to indicate the amount of minutes post glucose consumption at the time of sampling. Whole blood was collected in EDTA tubes containing aprotinin (50 KIU/mL blood; Becton Dickinson, Plymouth, UK). C‐Reactive protein (CRP) was measured from fasted venous blood at a commercial laboratory (Western Diagnostics Pathology, Myaree, WA, Australia). Blood glucose from OGTT samples was determined using a chemical analyser (COBAS Integra 400 plus, Roche Diagnostics Ltd, Switzerland). The area under the curve (AUC) was calculated for glucose concentrations from the OGTTs using the trapezoidal method (Graphpad Prism, RRID:SCR_002798).

#### 2.3.2 Intervention

The exercise intervention commenced on the day after the OGTT. Over the course of 12 sequential days, participants performed 45 min of supervised treadmill exercise, during which the workload was adjusted dynamically such that the heart rate of the individual reached the value associated with 65% of their ${\dot{V}}_{O_{2}\mathrm{peak}}$ as determined by their pre‐intervention incremental exercise test. Venous blood samples were obtained before (tp_pre_) and immediately after (tp_post_) the first (Ex1) and last (Ex12) training sessions. Participants were asked to maintain a consistent diet for the duration of the study period.

#### 2.3.3 Postintervention visits

At least 24 h and at most 48 h after the last training session, participants visited the exercise physiology laboratory to complete a second OGTT (OGTT 2) according to the same protocol and at the same time of day as during the pre‐intervention OGTT procedure. Participants were advised to replicate their meal in the 24 h preceding the second OGTT to match their food intake prior to the first OGTT. Diet was, however, at the discretion of participants and was not standardized. Finally, participants performed another incremental exercise test identical to the test protocol prior to the intervention.

### 2.4 Analytical methods

#### 2.4.1 Sample storage and analysis

After sampling, EDTA tubes were immediately centrifuged at 1300*g* for 10 min. Plasma samples were then aliquoted into triplicates and stored at −80°C pending further preparation and subsequent analyses. Samples of the experimental cohort were stored for ∼5 years prior to analysis, while samples of the external cohorts of T2DM‐controls and healthy controls were stored for periods ranging from a few months to 11 years. ^1^H NMR‐ and liquid chromatography–mass spectrometry (LC‐MS)‐based metabolic phenotyping was conducted at the Australian National Phenome Centre (Murdoch University).

#### 2.4.2 NMR: Sample preparation and analysis

^1^H NMR‐based metabolic phenotyping was performed to analyse lipoproteins, amino acids, glycoproteins and the supramolecular phospholipid composite (SPC) as reported previously by Dona and colleagues (Dona et al., 2014; Lodge et al., 2024, 2021). In brief, plasma centrifugation was followed by the collection of plasma supernatant, which was subsequently mixed with buffer [75 mM Na_2_HPO_4_, 2 mM NaN_3_, 4.6 mM sodium trimethylsilyl propionate‐[2,2,3,3‐^2^H_4_] (TSP) in 80% D_2_O, pH 7.4 ± 0.1] (1:1). Six hundred microlitres of the resulting plasma mixture was transferred to 5 mm Bruker SampleJet NMR tubes for NMR measurements. In‐house long‐term reference samples were prepared following the same procedures and were used as quality control (QC) samples. Two Bruker 600 MHz Avance III HD spectrometers equipped with a BBI probe and with integral Bruker SampleJet robots were used for all NMR measurements. The previously published Bruker in vitro diagnostics research (IVDr) methods were used to complete NMR measurements (Jiménez et al., 2018). NMR data were processed automatically using Bruker Topspin v.3.6.2 and ICON NMR to enable phasing, baseline correction and calibration to TSP (δ = 0).

#### 2.4.3 Lipid‐specific LC‐MS: Sample preparation and analysis

A previously published targeted lipidomic protocol for the detection of 1163 lipid species was modified regarding internal standard mixtures to be used for the analysis of plasma lipids (Ryan et al., 2023). Thawing of plasma samples was performed at 4°C, and 10 µL was aliquoted to 96‐well plates (Eppendorf, Macquarie Park, NSW, Australia). Ninety microlitres of propan‐2‐ol containing stable isotope labelled internal standards (SIL ISTDs, diluted to reach a concentration of 1:500) were added, followed by vortex mixing. The SIL ISTDs consisted of UltimateSPLASH ONE (Avanti Polar Lipids), SphingoSPLASH and 0.01 µg/mL MG 18:1‐d7 (Sigma‐Aldrich, North Ryde, NSW, Australia and 0.005 µg/mL arachidonic acid‐d5, linoleic acid‐d11, oleic acid‐d9, palmitic acid‐d5 and stearic acid‐d4 purchased from Cayman Chemical (Sapphire Bioscience, Redfern, NSW, Australia). Following the addition of the SIL ISTD‐IPA mixture to the sample wells, the Eppendorf plates were mixed at room temperature for 10 min at 14 000*g*. The supernatant was then transferred to a new 96‐well plate for LC‐MS analysis. Liquid chromatography was performed by a SCIEX ExionLC (SCIEX, Concord, CA, USA), where separation was conducted using Waters Acquity BEH C_18_ 1.7 µm, 2.1 mm × 100 mm column (Waters Corp., MA, USA) at 60°C. Mass detection was conducted by a SCIEX QTRAP 6500+ (SCIEX, Concord, CA, USA) with electrospray ionization and polarity switching. Samples were stored in the autosampler at 10°C, and the sample injection volume was 5 µL. SCIEX Analyst v.1.7.1 (SCIEX, MA, USA) software was used to direct LC‐MS operation and acquire data. Time‐scheduled multiple reaction monitoring (MRM) was used for the data acquisition of 1230 transitions (including 1163 lipid species and 67 internal standards).

Skyline software (v.21.1, MacCoss, WA, USA, RRID:SCR_014080) was used to integrate the lipid peaks analysed by LC‐MS and to calculate the target analyte response to internal standard ratios. Further data preprocessing was conducted in RStudio v.1.4.1 (R Foundation for Statistical Computing, Vienna, Austria). Features were filtered for missing values and were removed if >50% of missing values were presented. All other features were imputed by dividing the minimum value by two, followed by deleting features with >30% relative standard deviation (RSD) across the replicate QC plasma samples. The random forest method within the statTarget package was used to correct for signal drift induced during the analytical run.

### 2.5 Statistical analyses

The preprocessed metabolic data collected on ^1^H NMR and LC‐MS platforms were merged for the purpose of statistical modelling. An evidence‐based panel of metabolites relevant to type 2 diabetes was selected for statistical analysis and modelling based on the literature cited in two recent literature reviews (Jin & Ma, 2021; Shahisavandi et al., 2023). In addition to the previously reported lipoprotein main fractions, a comprehensive NMR lipoprotein measurement, including 91 lipoproteins and subclasses, was incorporated to expand the existing body of knowledge regarding lipoprotein concentrations in T2DM (Jiménez et al., 2018). Furthermore, SPC signals, in addition to glycoproteins A and B, were included, given their association with inflammation (Connelly et al., 2017a; Lodge et al., 2024). An overview of the selected metabolites is provided in Table A1.

The statistical analyses comprised a combination of multi‐ and univariate analyses. Univariately, the characteristics of participants from all groups were compared using Kruskal–Wallis and Dunn's *post hoc* testing with Bonferroni correction (https://github.com/kassambara/rstatix, RRID:SCR_021240). Multivariate approaches consider multiple outcome parameters (i.e., multiple metabolites) and additionally allow for the simultaneous establishment of correlational patterns between these parameters (Alonso et al., 2015; Saccenti et al., 2014), thus making them suitable methods for the comparison of two metabolic datasets comprising large volumes of analytes. Here, principal component analysis (PCA) was conducted to determine whether the plasma metabolome of participants with T2DM was different from that of healthy individuals and similar to that of T2DM participants from an independent cohort, prior to initiation of the study, and to explore whether the intervention resulted in the T2DM metabolome shifting away from that of T2DM‐controls and closer to the metabolome of healthy controls. As such, sample data from healthy controls and T2DM‐controls were analysed with PCA. Sample data from tp_0_ of OGTT 1 and OGTT 2 of the T2DM cohort taking part in the intervention were then centred, scaled, and projected onto the existing PCA model. The PCA results were visualized using a scores plot. Additionally, 25 metabolites commonly cited as relevant in T2DM were visualized in boxplots to highlight differences in concentrations between healthy controls, T2DM‐controls and T2DM participants before and after the intervention. Kruskal–Wallis tests with Bonferroni *p*‐value adjustment were used to assess group differences for each of these 25 metabolites. For significant group effects, Dunn's *post hoc* tests with Bonferroni correction were applied to determine which specific group comparisons contributed to the overall effect.

To investigate intervention‐induced alterations in the metabolic response to (1) an OGTT and (2) an acute exercise stimulus, repeated‐measures ANOVA simultaneous component analysis + (RM‐ASCA^+^) was conducted using the ALASCA package in R (Jarmund et al., 2022). The RM‐ASCA^+^ method allows for a combination of general linear (mixed) models with PCA and is particularly suitable for multivariate data from longitudinal studies, as is the case here. The RM‐ASCA^+^ models for analysis of the OGTT and exercise data were initialized as:

$$\mathrm{Metabolite}\;\mathrm{concentration}∼\mathrm{TP}\times \mathrm{tp}+\left(1|\mathrm{ID}\right)$$

The formula represents a linear mixed model with metabolite concentration as the outcome variable. TP refers to the pre‐ or postintervention OGTT (i.e., OGTT 1 and OGTT 2) and the first or last day of training for the exercise data (i.e., Ex1 and Ex12). tp denotes the TP of blood sample collection either during the OGTT (i.e, tp_0_, tp_30_, tp_60_, tp_90_ and tp_120_) or before and after the exercise session (i.e., tp_pre_ and tp_post_). ID is the participant identification number, which contributed to the model as a random effect. The interaction term TP × tp allowed for a comparison of the TPs within an OGTT and between the two OGTTs. Specifications of the ALASCA function included the scaling function, separate effects, validation, number of validation runs, validation method, dimension reduction, usage of Rfast, limits of the confidence intervals and *p*‐value adjustment method. The scale function was set to ‘sdt1’ to divide the values by the SD of all baseline samples, per metabolite. In that way, the values at tp_30_, tp_60_, tp_90_ and tp_120_ were standardized with reference to tp_0_, and the values at tp_post_ were standardized with reference to tp_pre_. ‘Separate effects = FALSE’ because there was only one group of participants for the OGTT data. ‘Validate’ was set to ‘TRUE’ to enable testing of the confidence of the estimated scores and loadings from RM‐ASCA^+^ by performing the analysis on 90 resampled data sets (as specified using ‘n_validation_runs’). Bootstrapping was chosen as ‘validation_method’, which randomly selected participants with replacement. PCA was performed by setting ‘reduce_dimensions’ to ‘TRUE’, and the lme4 package was used for linear mixed modelling by specifying ‘use_Rfast’ as ‘FALSE’. The upper and lower percentiles of the confidence intervals were set to 0.025 and 0.975, respectively. Finally, false discoveries were controlled for by specifying the ‘*p*_adjust_method’ as ‘fdr’.

A scree plot was created to determine the number of principal components requiring further investigation. For each principal component deemed relevant, the scores and loadings were visualized and prediction plots were created for the 15 metabolites with the highest and lowest loadings on each principal component of interest. All figures related to RM‐ASCA^+^ modelling were created using the ALASCA package (Jarmund et al., 2022).

All data and statistical analyses were performed in RStudio v.4.3.3 (R Foundation for Statistical Computing, Vienna, Austria, RRID:SCR_000432). Relevant packages included mva.plots, ALASCA and ggplot2 (Jarmund et al., 2022; Wickham, 2016; https://github.com/phenological/mva‐plots). The level of statistical significance was set to *p* ≤ 0.05.

## 3 RESULTS

### 3.1 Participant characteristics

Thirteen T2DM participants (eight females) met the inclusion criteria for the exercise intervention. The majority of participants were on oral hypoglycaemic medication (*n* = 12; i.e., metformin, sulfonylurea or a combination of both), and some took anti‐hypertensive (*n* = 4) and/or anti‐depressive (*n* = 2) drugs. All participants achieved 100% compliance with the exercise intervention. Owing to difficulties with blood sample collection before and immediately after exercise on the first and last day of the intervention programme, data were available for only nine participants (four male and five female) at these TPs. Blood samples were collected for all 13 participants taking part in the OGTT as planned.

Given that age differed significantly between groups (T2DM controls vs. healthy controls, *p *< 0.0001; T2DM controls vs. T2DM pre‐intervention, *p *< 0.0001; healthy controls vs. T2DM pre‐intervention, *p *= 0.037; Table 1), all data used to model differences between persons with T2DM taking part in the intervention, healthy controls and T2DM controls were corrected for age. Neither body mass nor body composition parameters were significantly altered in response to the short‐term exercise intervention (Table 1; body mass, *p *= 0.809; fat percentage, *p *= 0.051; VAT, *p *= 0.450). Additionally, neither fasted blood glucose nor the 2 h glucose AUC during the OGTT was significantly reduced (*p* = 0.301 and *p *= 0.135, respectively), nor was CRP (*p *= 0.079; Table 1). There was a marginal increase in ${\dot{V}}_{O_{2}\mathrm{peak}}$ following the exercise intervention (i.e., 23.1 ± 4.9 before vs. 24.1 ± 4.7 mL/kg/min after the intervention, *p *= 0.011; Table 1).

**TABLE 1:** Participant characteristics before and after the exercise intervention.

Parameter	T2DM participants		Healthy control group	T2DM control group
	Pre‐intervention	Postintervention		
*n*	13 (8 female)		198 (103 female)	208 (100 female)
Age, years	51 ± 7^**^, ^***^		57 ± 6^******^	63 ± 6
BMI, kg/m^2^	32.7 ± 4.9^**^	32.7 ± 4.6^****^	22.6 ± 1.7^******^	31.8 ± 2.7
Weight, kg	90.6 ± 14.8^**^	90.7 ± 14.6^****^	66 ± 10^******^	90 ± 12
Fat, %	37.1 ± 6.4	36.2 ± 7.1		
VAT, g	901.6 ± 340.3	925.8 ± 366.9		
HbA_1c_, %	7.35 ± 1.41^**^		5.47 ± 0.25^******^	6.65 ± 1.19
CRP, mg/L	5.3 ± 4.0^**^	4.1 ± 2.8^****^	1.4 ± 1.9^******^	4.3 ± 6.6
FBG, mmol/L	10.3 ± 3.9	9.8 ± 4.0		
2 h Glucose AUC, mmol/L min	1931 ± 514	1830 ± 590		
${\dot{V}}_{O_{2}\mathrm{peak}}$, mL/kg/min	23.1 ± 4.9^*^	24.1 ± 4.7		

*Note*: Data are presented as the mean ± SD. Significance was determined by Dunn's testing with Bonferroni correction. Exact *p*‐values for all comparisons are provided in Table A1.

Abbreviations: AUC, area under the curve; BMI, body mass index; CRP, C‐reactive protein; FBG, fasted blood glucose; HbA_1c_, haemoglobin A_1c_; T2DM, type 2 diabetes mellitus; VAT, visceral adipose tissue; ${\dot{V}}_{O_{2}\mathrm{peak}}$, peak oxygen uptake during exercise.

^*^ *p* < 0.05, T2DM participants pre‐ versus postintervention.

^**^ *p *< 0.05, T2DM participants pre‐intervention versus healthy control subjects.

^***^ *p* < 0.05, T2DM participants pre‐intervention versus T2DM control subjects.

^****^ *p *< 0.05, T2DM participants postintervention versus healthy control subjects.

^*****^ *p* < 0.05, T2DM participants postintervention versus T2DM control subjects.

^******^ *p *< 0.05, Healthy control subjects versus T2DM control subjects.

### 3.2 Quantified metabolites

Sample analysis with NMR returned the quantification of 143 small molecules, lipoproteins and subclasses plus inflammatory markers, and semi‐targeted LC‐MS provided measurement for 834 lipid species across 18 lipid sub‐classes. The combined data matrix thus consisted of 977 metabolic analytes, of which 241 were strategically selected for statistical analyses based on their relevance in T2DM according to the literature (Table A1). Of these 241, only the 118 metabolites measured by ^1^H NMR spectroscopy were used for the comparison between healthy controls and T2DM participants, because no lipidomics data were available from the healthy control group.

### 3.3 The metabolome of people living with type 2 diabetes is distinct from healthy controls before and after 12 days of training

**FIGURE 2:**
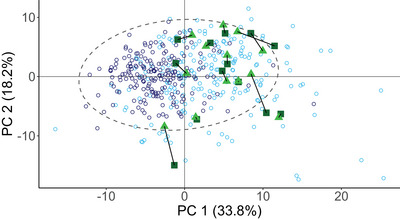
PCA scores plot, where the fasted metabolomes of healthy control subjects (dark blue open circles, *n* = 198) are compared with T2DM control subjects (pale blue open circles, *n *= 208) and where T2DM before (dark green squares, *n *= 13) and after (bright green triangles, *n *= 13) the intervention are projected onto the PCA model. T2DM pre‐ and postintervention scores are linked per participant. The dashed ellipse represents Hotellings *T* ^2^ 95% confidence around the scores for healthy and T2DM control subjects. The total expressed variance in the first and second principal components is 52%. Abbreviations: PCA, principal component analysis; T2DM, type 2 diabetes mellitus.

PCA containing all 118 NMR metabolites revealed some overlap between the fasted metabolic signature of healthy controls and T2DM‐controls, although the majority of the T2DM‐controls showed a signature vastly distinct from healthy controls (Figure 2). From the projection of T2DM participants taking part in the exercise training programme in this study onto this PCA model, it can be seen that their metabolome is more similar to that of T2DM‐controls than to healthy controls (Figure 2). People with T2DM who took part in the intervention did not shift towards a healthy metabolic profile, because most postintervention scores are still closer to the scores of T2DM‐controls than to those of healthy controls, indicating that metabolite values for T2DM participants were still considerably different from healthy values after the exercise intervention. From the PCA scores, it became clear that the first principal component (PC1) is responsible for a large portion of the variance in the dataset (i.e., 33.8%) and primarily drove the separation between T2DM‐controls and healthy controls (Figure 2). The metabolites most influential in driving this separation can be derived from the loadings of PC1 and the second principal component (PC2), which are provided in Table A3.

**FIGURE 3:**
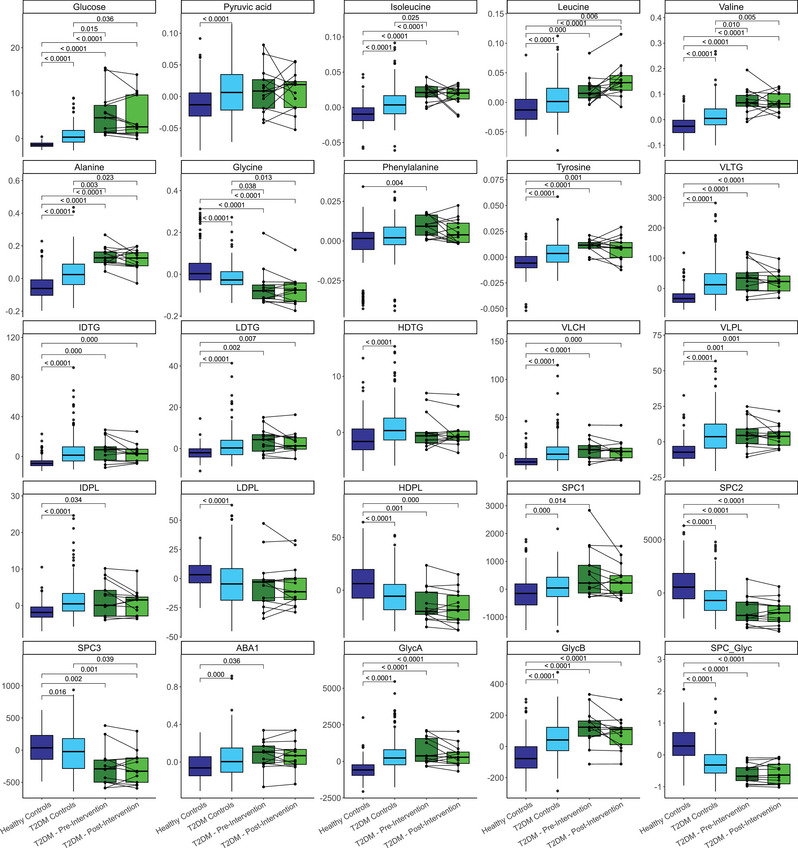
Boxplots of 25 metabolites commonly reported to be altered in T2DM. Pre‐ and postintervention values of T2DM participants taking part in the exercise training programme (*n *= 13) are contrasted against healthy control subjects (*n *= 198) and T2DM control subjects (*n *= 208). T2DM participants who took part in the intervention are paired by their pre‐ and postintervention values. Abbreviations: ABA1, apolipoprotein B100/apolipoprotein A1 ratio; GlycA, glycoprotein A; GlycB, glycoprotein B; HDPL, high density lipoprotein (HDL) phospholipid; HDTG, HDL triglyceride; IDPL, intermediate density lipoprotein (IDL) phospholipid; IDTG, IDL triglyceride; LDPL, low density lipoprotein (LDL) phospholipid; LDTG, LDL triglyceride; SPC1, supramolecular phospholipid composite 1; SPC2, supramolecular phospholipid composite 2; SPC3, supramolecular phospholipid composite 3; SPC_Glyc, supramolecular phospholipid composite/glycoprotein ratio; T2DM, type 2 diabetes mellitus; VLCH, very low density lipoprotein (VLDL) cholesterol; VLPL, VLDL phospholipid; VLTG, VLDL triglyceride. Metabolite values are presented in the following units: small molecules (in millimoles per litre); lipoproteins (in milligrams per decilitre); SPCs, Glycs and ABA1 (in arbitrary units). Significance was determined by Dunn's *post hoc* testing with Bonferroni correction. Only significant contrasts are shown.

A selected panel of 25 metabolites commonly reported as altered in T2DM was displayed with boxplots (Figure 3). Glucose (*p *< 0.0001), the BCAAs isoleucine (*p *< 0.0001), leucine (*p *= 0.000) and valine (*p *< 0.0001), amino acids alanine (*p *< 0.0001), phenylalanine (*p *= 0.004) and tyrosine (*p *< 0.0001), in addition to very low density lipoprotein (VLDL) triglyceride (*p *< 0.0001; VLTG), cholesterol (*p *< 0.0001; VLCH) and phospholipid (*p *= 0.001; VLPL), low density lipoprotein (LDL) triglyceride (*p *= 0.002; LDTG), intermediate density lipoprotein (IDL) triglyceride (*p *= 0.000; IDTG) and phospholipid (*p *= 0.034; IDPL), SPC 1 (*p *= 0.014; SPC1), apolipoprotein B100/apolipoprotein A1 ratio (*p *= 0.036; ABA1) and glycoproteins A (*p *< 0.0001; GlycA) and B (*p *< 0.0001; GlycB) were significantly higher in T2DM when compared with healthy controls, as expected (Figure 3). Glycine (*p *< 0.0001), high density lipoprotein (HDL) phospholipid (*p *= 0.001; HDPL), SPC2 (*p *< 0.0001), SPC3 (*p *= 0.002) and the SPC/Glyc ratio (*p *< 0.0001) (Figure 3) were lower in T2DM when compared with healthy controls. Most metabolite concentrations were similar between T2DM‐controls and T2DM participants taking part in the exercise programme, consistent with their selection for the presence of T2DM. The pre‐ to postintervention changes in metabolite concentrations were inconsistent between individuals, with some individuals showing a decreased postintervention concentration relative to their pre‐intervention concentration, but others showing an increase.

### 3.4 Metabolic response to oral glucose is marginally altered after a 12 day exercise intervention

**FIGURE A1:**
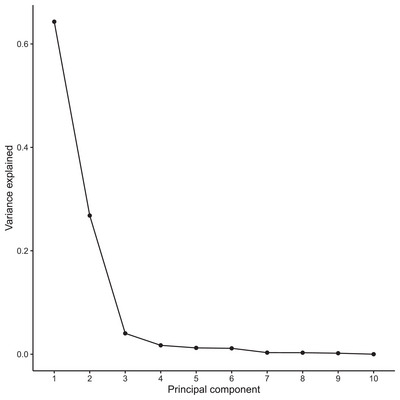
Scree plot of the RM‐ASCA^+^ model with the OGTT data, showing the amount of variance explained by each principal component. Abbreviations: OGTT, oral glucose tolerance test.

**FIGURE A2:**
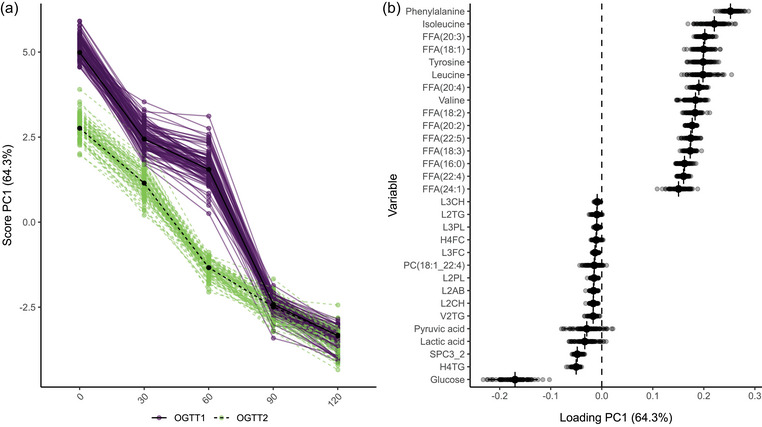
Validation scores (a) and loadings (b) plots of the RM‐ASCA^+^ model for the OGTT data displaying the scores and loadings of each bootstrap iteration for T2DM participants taking part in the exercise intervention (*n* = 13). Green and purple lines represent individual bootstrap iterations, and black lines (continuous black line for OGTT 1 and dashed black line for OGTT 2) represent the main model (a). Abbreviations: FFA, free fatty acid; HxFC*, free cholesterol high density lipoprotein (HDL) subfraction; HxTG*, triglyceride HDL subfraction; LxAB*, apolipoprotein B low density lipoprotein (LDL) subfraction; LxCH*, cholesterol LDL subfraction; LxFC*, free cholesterol LDL subfraction; LxPL*, phospholipid LDL subfraction; LxTG*, triglyceride LDL subfraction; OGTT, oral glucose tolerance test; PC, phosphatidylcholine; PC1, first principal component; SPC3_2, supramolecular phospholipid composite 3/supramolecular phospholipid composite 2 ratio; VxTG*, triglyceride very low density lipoprotein (VLDL) subfraction. *Where x is a number from 2 to 4.

The RM‐ASCA^+^ model was built using the OGTT time points (i.e., 30, 60, 90 and 120 min) collected during the pre‐ and postintervention laboratory visits. The RM‐ASCA^+^ model revealed two overall patterns of change, with PC1 and PC2 explainin 64.30% and 26.81% of the variance in the data, respectively (Figure A1). The scores and loadings for each bootstrap iteration, in addition to the confidence intervals of the loadings, are shown in Figure A2.

**FIGURE 4:**
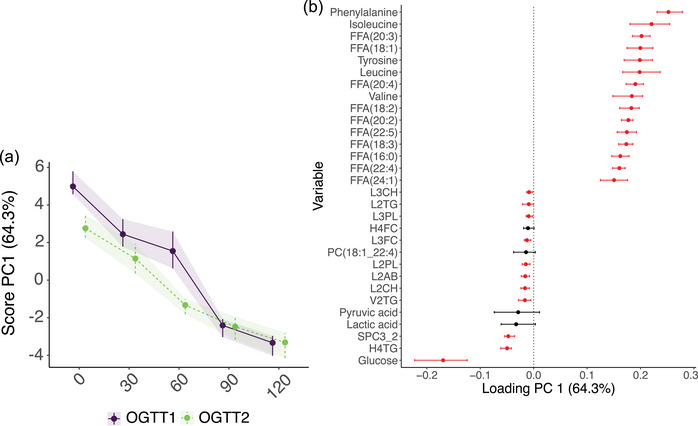
Development of the metabolome over time during OGTT 1 (purple) and OGTT 2 (green) of T2DM participants taking part in the exercise intervention (*n *= 13). Scores (a) and loadings (b) of the first principal component are displayed. Metabolites with positive loadings follow the time trajectory as shown in the scores plot, increasing and decreasing when the scores increase and decrease, respectively, whereas metabolites with negative loadings follow the opposite trajectory over time. Abbreviations: FFA, free fatty acid; H4FC, free cholesterol high density lipoprotein (HDL) subfraction; H4TG, triglyceride HDL subfraction; L2AB, apolipoprotein B low density lipoprotein (LDL) subfraction; L2CH, cholesterol LDL subfraction; L2TG, triglyceride LDL subfraction; L2PL, phospholipid LDL subfraction; L3CH, cholesterol LDL subfraction; L3FC, free cholesterol LDL subfraction; L3PL, phospholipid LDL subfraction; OGTT, oral glucose tolerance test; PC, phosphatidylcholine; PC1, first principal component; SPC3_2, supramolecular phospholipid composite 3/supramolecular phospholipid composite 2 ratio; T2DM, type 2 diabetes mellitus; V2TG, triglyceride very low density lipoprotein (VLDL) subfraction.

Metabolites with positive loadings on PC1 of the RM‐ASCA^+^ model (Figure 4) decreased over time in both OGTT 1 and OGTT 2, whereas metabolites with negative loadings on PC1 increased over time. Phenylalanine and glucose showed the largest decrease and increase over time because they have the highest and lowest positive and negative loadings, respectively. From the parallel and partial overlap of the two OGTT line trajectories over time, it can be derived that PC1 mainly explains the variance between TPs within the OGTTs, rather than differences between the two OGTTs.

**FIGURE 5:**
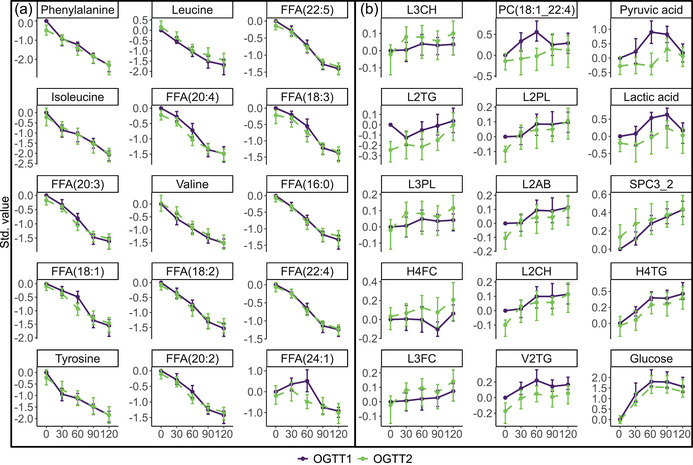
Scaled plasma metabolite trajectories over the time course of OGTT 1 and OGTT 2 for individuals with T2DM taking part in the exercise intervention (*n *= 13). (a) Metabolites with positive loadings on the first principal component as shown in Figure 4b. (b) Metabolites with negative loadings on the first principal component as shown in Figure 4b. Abbreviations: FFA, free fatty acid; H4FC, free cholesterol high density lipoprotein (HDL) subfraction; H4TG, triglyceride HDL subfraction; L2AB, apolipoprotein B low density lipoprotein (LDL) subfraction; L2CH, cholesterol LDL subfraction; L2TG, triglyceride LDL subfraction; L2PL, phospholipid LDL subfraction; L3CH, cholesterol LDL subfraction; L3FC, free cholesterol LDL subfraction; L3PL, phospholipid LDL subfraction; OGTT, oral glucose tolerance test; PC, phosphatidylcholine; SPC3_2, supramolecular phospholipid composite 3/supramolecular phospholipid composite 2 ratio; V2TG, triglyceride very low density lipoprotein (VLDL) subfraction.

For metabolites with the largest positive and negative loadings as displayed in Figure 4b, the scaled metabolite concentrations over time during both OGTTs are shown in Figure 5. These plots confirm that metabolites with positive loadings decrease over time and follow a highly similar trajectory in OGTT 1 and OGTT 2 (Figure 5a). On the contrary, some of the metabolites with negative loadings [including L2TG, PC(18:1_22:4), V2TG, pyruvic acid, lactate and H4TG] had somewhat higher metabolite levels in OGTT 1 compared with OGTT 2, indicated by the line trajectory of OGTT 1 being higher than that of OGTT 2 (Figure 5b).

**FIGURE A3:**
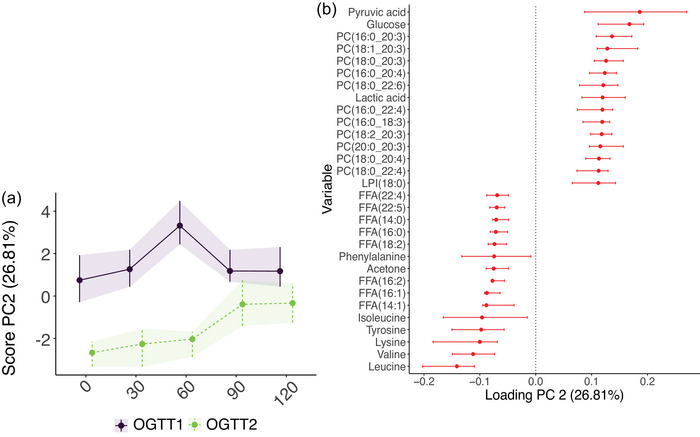
Development of the metabolome over time during OGTT 1 (purple) and OGTT 2 (green) for individuals with T2DM participating in the exercise intervention (*n* = 13). Scores (a) and loadings (b) of the second principal component are displayed. Metabolites with positive loadings follow the time trajectory as shown in the scores plot, increasing and decreasing when the scores increase and decrease, respectively, whereas metabolites with negative loadings follow the opposite trajectory over time. Abbreviations: FFA, free fatty acids; LPI, lysophosphatidylinositol; OGTT, oral glucose tolerance test; PC, phosphatidylcholine; PC2, second principal component.

**FIGURE A4:**
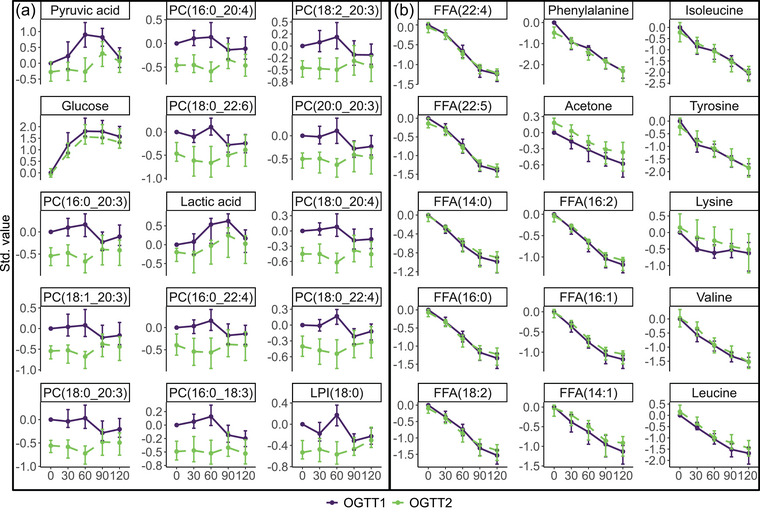
Scaled metabolite trajectories over the time course of OGTT 1 and OGTT 2 for T2DM participants taking part in the exercise intervention (*n* = 13). (a) Metabolites with positive loadings on the second principal component as shown in Figure A3b. (b) Metabolites with negative loadings on the second principal component as shown in Figure A3b. Abbreviations: FFA, free fatty acids; LPI, lysophosphatidylinositol; OGTT, oral glucose tolerance test; PC, phosphatidylcholine; T2DM, type 2 diabetes mellitus.

From the scores and loadings of PC2 (Figure A3), it can be observed that metabolites with positive loadings peaked at tp_60_ of OGTT 1, but not at tp_60_ of OGTT 2. Indeed, the scaled trajectories of the individual metabolites with highest positive loadings on PC2 confirm that the glycolysis‐related metabolite pyruvic acid, in addition to various phosphatidylcholines and one lysophosphatidylinositol, peaked at tp_60_ of the first OGTT (Figure A4a). The standardized values of these metabolites over the time course of a glucose test are clearly separated between OGTT 1 and OGTT 2. Metabolites with the largest negative loadings on PC2 (Figure A4b) are largely similar to those with positive loadings on PC1 and are discussed above.

### 3.5 Metabolic signatures of acute exercise stimuli followed the same trajectory at a lower level after a 12 day exercise programme

**FIGURE A5:**
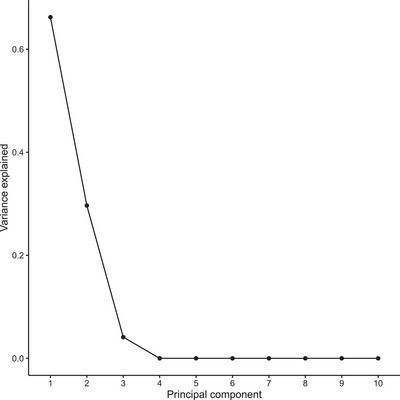
Scree plot of the RM‐ASCA^+^ model with the exercise data, showing the amount of variance explained by each principal component.

**FIGURE A6:**
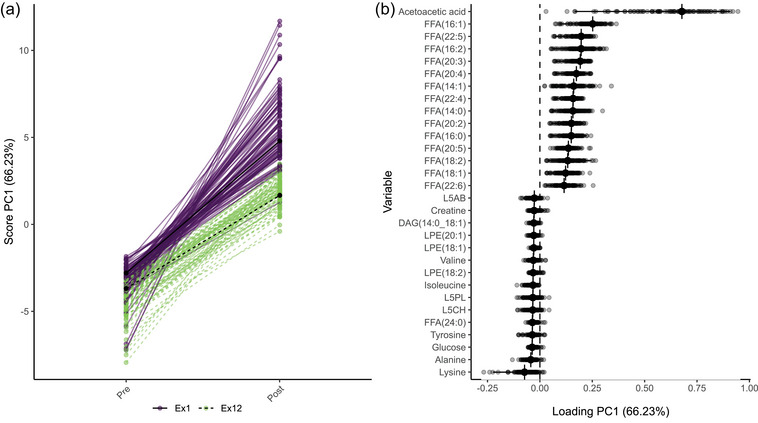
Validation scores (a) and loadings (b) plots of the RM‐ASCA^+^ model for the exercise data displaying the scores and loadings of each bootstrap iteration for people with T2DM who participated in the exercise intervention and whose samples could be collected before and after the first and last training sessions (*n* = 9). Green and purple lines represent individual bootstrap iterations, and the black lines (continuous black line for Ex1 and dashed black line for Ex2) represent the main model (a). Abbreviations: DAG, diacylglycerol; FFA, free fatty acid; L5CH, cholesterol low density lipoprotein (LDL) subfraction; L5PL, phospholipid LDL subfraction; LPE, lysophosphatidylethanolamine; PC1, first principal component; T2DM, type 2 diabetes mellitus.

The scree plot related to the RM‐ASCA^+^ model constructed with the metabolite data of blood plasma samples collected before and after the first and last training session revealed two main patterns of variation, as indicated by the amount of variance explained by PC1 (66.23%) and PC2 (29.63%) (Figure A5). In the RM‐ASCA^+^ validation scores plot, a large variety in pre‐ and postexercise scores exist among the individual bootstrap iterations (Figure A6a), indicating variance within metabolites. Likewise, the confidence intervals of the loadings are large, particularly for acetoacetic acid and lysine (Figure A6b).

**FIGURE 6:**
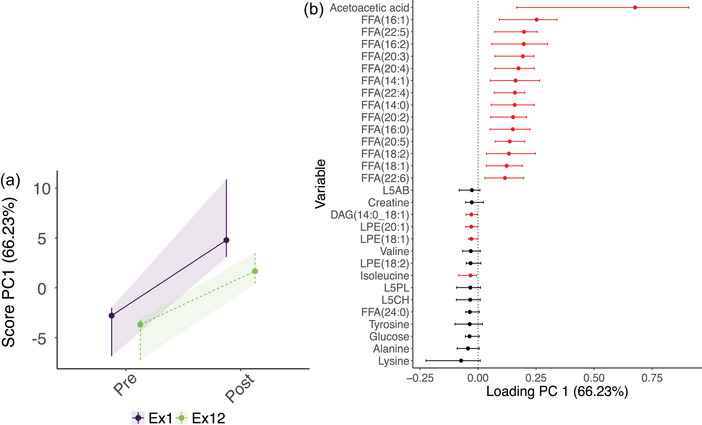
Development of the metabolome over time during the first (purple) and last (green) exercise training for people with T2DM who participated in the exercise intervention and whose samples could be collected before and after the first and last training sessions (*n *= 9). Scores (a) and loadings (b) of the first principal component are displayed. Metabolites with positive loadings follow the time trajectory as shown in the scores plot, increasing and decreasing when the scores increase and decrease, respectively, whereas metabolites with negative loadings follow the opposite trajectory over time. Abbreviations: DAG, diacylglycerol; FFA, free fatty acid; L5CH, cholesterol low density lipoprotein (LDL) subfraction; L5PL, phospholipid LDL subfraction; LPE, lysophosphatidylethanolamine; PC1, first principal component; T2DM, type 2 diabetes mellitus.

**FIGURE 7:**
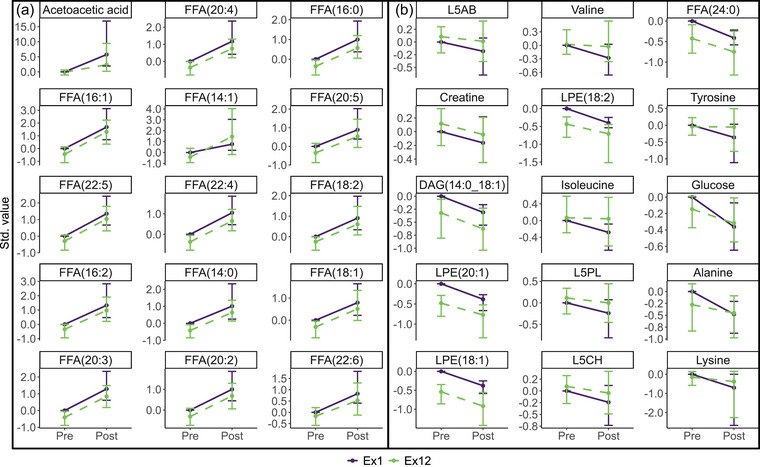
Scaled plasma metabolite trajectories over the time course of the first and last exercise training session for people with T2DM who participated in the exercise intervention and whose samples could be collected before and after the first and last training sessions (*n *= 9). (a) Metabolites with positive loadings on the first principal component as shown in Figure 6b. (b) Metabolites with negative loadings on PC1 as shown in Figure 6b. Abbreviations: DAG, diacylglycerol; FFA, free fatty acid; L5CH, cholesterol low density lipoprotein (LDL) subfraction; L5PL, phospholipid LDL subfraction; LPE, lysophosphatidylethanolamine; T2DM, type 2 diabetes mellitus.

Figure 6 presents a visualization of the PC1 scores and loadings of the RM‐ASCA^+^ exercise model. The confidence intervals of the scores for the first and last exercise session overlap, denoting a stronger effect of the acute exercise stimulus than the effect of the 12 day training intervention to change the response to acute exercise. Therefore, PC1 is responsible for distinguishing pre‐ and postexercise scores from each other rather than pre‐ versus postintervention scores. Free fatty acids follow the same trajectory over time, with most of the FFAs having somewhat lower levels at pre‐ and postexercise following the 12‐day training intervention when compared with before the intervention (Figure 6a). As opposed to acetoacetic acid and FFAs, several amino acids and lysophosphatidylethanolamines decreased in response to acute exercise on the first and last days of training (Figures 6b and 7).

**FIGURE A7:**
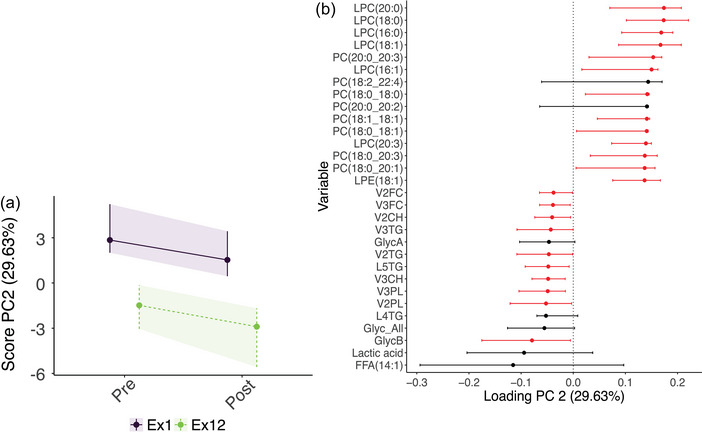
Development of the metabolome over time during the first (purple) and last (green) exercise training for people with T2DM who participated in the exercise intervention and whose samples could be collected before and after the first and last training sessions (*n* = 9). Scores (a) and loadings (b) of the second principal component are displayed. Metabolites with positive loadings follow the time trajectory as shown in the scores plot, increasing and decreasing when the scores increase and decrease, respectively, whereas metabolites with negative loadings follow the opposite trajectory over time. Abbreviations: FFA, free fatty acids; GlycA, glycoprotein A; Glyc_All, glycoprotein total; GlycB, glycoprotein B; LPC, lysophosphatidylcholine; LPE, lysophosphatidylethanolamine; Lx*TG, triglyceride low density lipoprotein (LDL) subfraction; PC, phosphatidylcholine; PC2, second principal component; T2DM, type 2 diabetes mellitus; Vx*CH, cholesterol VLDL subfraction; Vx*FC, free cholesterol VLDL subfraction; Vx*PL, phospholipid very low density lipoprotein (VLDL) subfraction; Vx*TG, triglyceride VLDL subfraction. *With x being a number from 2 to 5.

**FIGURE A8:**
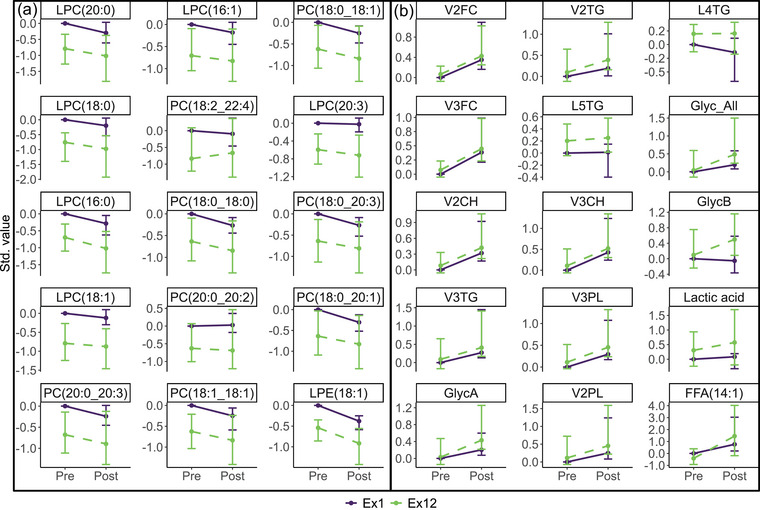
Scaled metabolite trajectories over the time course of the first and last exercise training session for T2DM participants who took part in the exercise intervention and whose samples could be collected before and after the first and last training sessions (*n* = 9). (a) Metabolites with positive loadings on the second principal component as shown in Figure A7b. (b) Metabolites with negative loadings on PC2 as shown in Figure A7b. Abbreviations: FFA, free fatty acids; GlycA, glycoprotein A; Glyc_All, glycoprotein total; GlycB, glycoprotein B; LPC, lysophosphatidylcholine; LPE, lysophosphatidylethanolamine; Lx*TG, triglyceride low density lipoprotein (LDL) subfraction; PC, phosphatidylcholine; Vx*CH, cholesterol VLDL subfraction; Vx*FC, free cholesterol very low density lipoprotein (VLDL) subfraction; Vx*PL, phospholipid VLDL subfraction; Vx*TG, triglyceride VLDL subfraction. *With x being a number from 2 to 5.

In contrast to PC1, PC2 underlies the separation between the metabolite scores of the first and last training session, as indicated by clearly divided lines (Figure A7a). Parallel lines indicate a stable difference between Ex1 and Ex12 for the duration of the exercise session. From the loading plot, it can be derived that various lysophosphatidylcholines and phosphatidylcholines, in addition to one of the lysophosphatidylethanolamines, showed a decrease in response to an acute exercise stimulus, with higher levels at Ex1 compared with Ex12 (Figure A7b). This finding is supported by the individual metabolite trajectories over the course of the first and last training session (Figure A8a). Metabolites with negative loadings on PC2 demonstrate an increase in response to acute exercise, with many of them displaying highly similar pre‐ and postexercise levels (i.e., free cholesterol and cholesterol VLDL subfractions, VLDL phospholipid subfractions and two VLDL triglyceride subfractions; Figure A8b). Lactate showed slightly elevated levels pre‐exercise, in addition to a larger increase on the final day of training compared with the first day of training.

## 4 DISCUSSION

We found that participants with T2DM had no improvements in body fat and glycaemic parameters (fasted blood glucose and 2 h glucose AUC) after 12 consecutive days of treadmill exercise for 45 min at ∼65% of ${\dot{V}}_{O_{2}\mathrm{peak}}$. To improve our understanding of the effects of a short‐term exercise intervention on the biochemical response to an OGTT and to an acute exercise challenge in people with T2DM and to discover whether the intervention shifted the biochemical signature of T2DM participants away from a typical T2DM profile and towards a healthier profile, blood samples were analysed using targeted metabolic profiling. Rather than examining the global metabolome, we concentrated on a strategically selected panel of key metabolites known to be relevant in the context of T2DM. We did not find beneficial effects of the exercise intervention on this targeted fasted metabolite profile or on the metabolic responses to an OGTT or to acute exercise in T2DM.

### 4.1 Type 2 diabetes versus healthy controls

PCA revealed similarities between the biochemical profile of T2DM subjects taking part in the present exercise training programme and T2DM‐controls (Figure 2). Although the PCA scores showed some overlap between healthy and T2DM‐controls, a large portion of individuals from both groups show distinct profiles. Using a predetermined panel of metabolites selected from previous research (Jin & Ma, 2021; Shahisavandi et al., 2023), it confirmed increased BCAAs (leucine, isoleucine and valine), alanine, phenylalanine and tyrosine, VLDL triglycerides, cholesterol and phospholipids, in addition to an elevated apolipoprotein B100/apolipoprotein A1 ratio in T2DM relative to the healthy control cohort (Figure 3). BCAAs have consistently been reported to be elevated in people with T2DM and to be correlated with insulin resistance (reviewed and reported by Jin & Ma, 2021; Shahisavandi et al., 2023; Vanweert et al., 2022). It is incompletely understood why this correlation exists and whether elevated levels of BCAAs induce insulin resistance or are biomarkers of T2DM (Cuomo et al., 2022). It has been suggested that the insufficient inhibition of protein breakdown, attributable to insulin insensitivity and dysfunctional BCAA catabolism, might explain higher concentrations of these amino acids in T2DM (Vanweert et al., 2022). VLDL particles, in particular triglyceride‐rich VLDL, are central to the lipid and lipoprotein abnormalities associated with hepatic dysfunction in T2DM and are increased in both fasting and postprandial states in individuals with T2DM (Søndergaard et al., 2017; Sørensen et al., 2011). The apolipoprotein B100/apolipoprotein A1 ratio is positively associated with T2DM prevalence (i.e., higher levels of apolipoprotein B100 and lower levels of apolipoprotein A1), owing to a dysregulation in the mechanistic effects of apolipoprotein B100 to inhibit lipolysis and/or apolipoprotein A1 to increase the uptake of glucose into skeletal muscle tissue and exert anti‐inflammatory actions (Gao et al., 2021). Apolipoprotein B is a carrier molecule for LDL, VLDL and IDL (Borén et al., 2020; Devaraj et al., 2024; Stanciulescu et al., 2023), which are significant risk factors for development of atherosclerotic cardiovascular disease (Borén et al., 2020; Stanciulescu et al., 2023), whereas apolipoprotein A carries HDLs, known for their anti‐thrombotic, anti‐inflammatory and antioxidative effects (van der Vorst, 2020). Elevated GlycA and GlycB in combination with decreased SPC2, SPC3 and SPC/Glyc are consistent with the notion that (overweight) people with diabetes present with chronic low‐grade systemic inflammation (Berbudi et al., 2020), given that these markers have previously been associated with inflammation (Lodge et al., 2024). Additionally, GlycA has been reported to be correlated positively with BMI and insulin resistance (Connelly et al., 2017b). Given the average BMI of the present study population (i.e., 32.7 kg/m^2^) and their T2DM diagnosis, it is therefore not surprising that GlycA is upregulated in this cohort when contrasted with healthy control subjects. Consistent with apolipoprotein A1 levels, HDL phospholipids were lower in T2DM subjects than in healthy controls (Figure 3). Low levels of HDLs are part of the dyslipidaemic profile associated with T2DM, the pathophysiological mechanisms of which have been reviewed elsewhere (Krauss, 2004).

Figure 3 shows distinct individual responses, with some individuals having increased levels of particular metabolites after the intervention, whereas other individuals had decreased or similar levels of particular metabolites after the intervention. For instance, some of the T2DM participants had reduced postintervention glucose levels, thus more closely resembling the median of the healthy controls, whereas others had even higher glucose levels postintervention (Figure 3). This might be attributable to the heterogeneity in individual responses to exercise‐based interventions, which is influenced by (epi)genetic and metabolic differences at baseline (Brennan et al., 2020). However, we cannot discount the possibility that these differences were based on intra‐individual variation in diet and/or other lifestyle factors over the course of the intervention. Given that the present study did not incorporate a cross‐over design or separate non‐exercising control group, the true reason for the variations between the pre‐ and postintervention fasted samples can therefore not be elucidated.

### 4.2 The metabolic response to an OGTT before and after 12 days of exercise in T2DM

Given that many people spend a significant portion of their time in a postprandial state, it is of importance to study the metabolome of people with T2DM not only in a fasting state, but also beyond (Gao et al., 2022; Wang et al., 2019). The metabolic response to the ingestion of 75 g of glucose included an increase in the glycolysis‐related metabolites glucose, pyruvic acid and lactate, but a decrease in the amino acids phenylalanine, isoleucine, leucine, tyrosine, lysine and valine, in addition to various FFAs (Figures 4 and 5). The glucose trajectory over the course of an OGTT is a hallmark of T2DM, with increasing levels until 60 min after bolus consumption, followed by a plateau at tp_90_ and a slight decrease towards tp_120_ (Figure 5b). This trajectory is different from that of healthy individuals, whose glucose levels already peak 30 min post‐consumption and decrease steeply afterwards, returning to baseline values at the 2 h mark (Wang et al., 2019). The glucose curve of T2DM participants in this study even shows some distinction from the curve of individuals with newly diagnosed type 2 diabetes (Wang et al., 2019), indicating a potential influence of disease duration on glucose tolerance.

In line with previous findings, BCAAs and aromatic amino acids (phenylalanine and tyrosine) decreased in response to the glucose bolus, which was previously explained by the action of insulin in suppressing proteolysis (Bentley‐Lewis et al., 2014; Gao et al., 2022; Wang et al., 2019). More specifically, ingestion of a glucose dose stimulates the release of insulin, which acts to suppress proteolysis and thus leads to lower levels of amino acids. In a similar fashion, insulin inhibits lipolysis, resulting in lower levels of FFAs. It is possible that this decrease is less pronounced or slower in the present study population than in healthy control subjects, which was the case in a previous study that compared the results of an OGTT between people with T2DM and those with normal glucose tolerance (Laws et al., 1997), but this was not investigated in the present study because no OGTT was conducted in a healthy population. Interestingly, pyruvic acid and lactate were consistently lower during OGTT 2 as compared with OGTT 1 (Figure A4). The reduction in pyruvate and lactate are consistent with a reduced reliance on the indirect pathway during glycogen resynthesis after the exercise training period (Kurland & Pilkis, 1989), which might have resulted from the exercise training‐induced adaptations, such as increased glycogen storage capacity and mitochondrial capacity. Lipid species of the class phosphatidylcholines and lysophosphatidylinositol (18:0) were both reduced in response to the exercise training intervention, as indicated by the consistently lower concentrations at all TPs during the OGTT. Increases in lysophosphatidylinositol (Jiménez‐Sánchez et al., 2024) and phosphatidylcholines, particularly the 20:3 classes, are linked with increased T2DM incidence (Prada et al., 2023). Therefore, the consistent decreases in the circulating concentrations of these lipid species in response to the exercise training would be expected to be beneficial. To our knowledge, exercise training‐induced changes in the concentration of lysophosphatidylinositols or phosphatidylcholines have not been assessed previously in T2DM.

### 4.3 The metabolic response to a training stimulus on the first and last day of a 12 day exercise intervention in people with T2DM

Targeted metabolite profiling data obtained from samples collected before and after the first and last training sessions revealed that exercise induced increases in acetoacetic acid and FFAs, in addition to decreases in glucose, the amino acids lysine, alanine, tyrosine, isoleucine and valine, creatine and three lysophosphatidylethanolamines (Figures 6 and 7). Participants exercised for 45 min at a heart rate associated with 65% of their ${\dot{V}}_{O_{2}\mathrm{peak}}$ during all training sessions, an exercise intensity and duration that predominantly demands the generation of ATP in aerobic conditions (Hargreaves & Spriet, 2020). In aerobic circumstances, the body uses a combination of carbohydrate and fat as substrates for energy; the relative contributions at different exercise intensities have been reviewed elsewhere (Hargreaves & Spriet, 2020; Muscella et al., 2020). During aerobic exercise at an intensity of 65% of the maximal oxygen uptake, FFAs provide a substantial amount of the total energy generated (Hargreaves & Spriet, 2020). Free fatty acids are released through the hydrolysis of triacylglycerols in adipose tissue and are transported into the mitochondria of muscle cells, where they generate ATP through the β‐oxidation pathway (Hargreaves & Spriet, 2020; Mika et al., 2019). The observed increases in FFAs postexercise relative to the pre‐exercise levels can thus be explained by elevated lipolysis, generating FFAs to be oxidized in muscle cells for ATP production. Related to this observation, acetoacetic acid, one of the three ketone bodies, increased considerably with exercise. Free fatty acids serve as the primary substrate for ketogenesis in the liver, and the observed increase in FFAs could thus be related to the increase in acetoacetic acid (Evans et al., 2017; Kolb et al., 2021). Exercise also has the capacity to induce the uptake of ketone bodies for oxidation and energy production in skeletal muscle (Pinckaers et al., 2017), although it appears that exercise‐induced production of acetoacetic acid exceeded consumption in the present population.

Remarkably, pre‐exercise levels of glucose were lower and postexercise levels higher on the last day of training in comparison to the first, which might indicate reduced uptake/usage of this metabolite during the last training session compared with the first (Figure 7b), although it cannot be ruled out that pre‐exercise values were altered owing to dietary intake. In line with the literature, isoleucine and valine decreased with acute exercise. Exercise‐induced reductions in circulating BCAAs were previously reported to indicate amino acid catabolism for energy production (Contrepois et al., 2020; Henriksson, 1991). However, previous research reported an accumulation of alanine and tyrosine in response to exercise owing to elevated cellular metabolism, whereas the present study found an opposite trend (Contrepois et al., 2020). It should be noted that participants in the cited study consisted of insulin‐sensitive and insulin‐resistant individuals, but not people living with type 2 diabetes, who exercised until exhaustion (Contrepois et al., 2020), whereas participants in the present study had confirmed T2DM and performed submaximal exercise. Insulin‐sensitive individuals showed a more severe increase in alanine than insulin‐resistant individuals (Contrepois et al., 2020), offering a potential explanation for the conflicting results of the present study.

The inflammatory markers GlycA and GlycB, predominantly from α‐1‐acid glycoprotein (Bell et al., 1987), had similar pre‐exercise levels but higher postexercise levels on the last day of the intervention than on the first. Glycoproteins were previously shown to be surrogate markers for CRP (Levine et al., 2020), a well‐known marker of inflammation. Elevated postexercise levels of GlycA and GlycB thus suggest a larger inflammatory response to exercise.

### 4.4 Limitations and future research

Although efforts were made to minimize any negative impact on the study outcomes, several limitations should be noted. The study population was small, with OGTT and exercise samples being collected in 13 and 9 participants, respectively. Given that the study adopted a repeated‐measures design, any statistically significant findings were dependent on within‐subject comparisons, except for the comparison of the pre‐ and postintervention fasted samples to healthy controls and T2DM‐controls (the latter two of which only NMR data was available of). This type of study design requires enough participants to reveal meaningful results (Gertsman & Barshop, 2018), and the small sample size might have resulted in insufficient statistical power for some of the comparisons. Nonetheless, similar research on the effects of training in a cohort of T2DM participants also included a small population (*n *= 15) (Savikj et al., 2022). The present study did not incorporate control conditions for the exercise intervention, and it can therefore not be ruled out whether mere inclusion in the study affected diet or other lifestyle choices that might have confounded the analyses. Changes in the metabolome observed in the exercise group might thus not be representative of the true effect of training. The inclusion of both a healthy and T2DM control group is a strength of this study because it allowed for an assessment of the effects of the intervention on inducing changes towards a healthy metabolic signature and away from a typical T2DM signature more objectively.

The lack of a dietary programme within the study design is considered a limitation and might have resulted in intra‐ and/or inter‐individual variation in pre‐ versus postintervention changes in the fasted metabolome, because diet has been shown to be one of the main parameters related to inter‐individual differences in the plasma metabolic signature (Chen et al., 2022). However, the gut microbiome and genetics are two other parameters strongly associated with variation in the metabolome between individuals (Chen et al., 2022), hence differences between participants would arguably still have existed even if diet were accounted for. Diet was not controlled prior to the exercise training sessions where samples were collected. To account for any potential pre‐exercise differences, postexercise values were scaled to pre‐exercise values, thus allowing for the investigation of the true metabolic response to exercise and differences herein between the first and last day of training. However, we cannot completely discount the potential variation in the metabolomic response being associated with differences in dietary intake. A strength of the study is that sessions were repeated at the same time of the day.

Although this study was focused on identifying biochemical changes in the early responses to exercise training (i.e., even before some clinical changes are evident), future research should compare these responses during both short‐ and medium‐term (e.g., 12 week) intervention periods. Additional exercise factors might then also be incorporated, including different durations and intensities, in addition to different exercise modalities (i.e., aerobic, anaerobic and resistance) on the same outcomes under investigation in the present study. This will allow for a comparison of the effectiveness of each protocol in improving the metabolic characteristics of T2DM for the investigation of a potential cut‐off beyond which no further improvements are observed.

Strengths in the design of the present study are that session durations of the present exercise programme complied with recommendations from the American Diabetes Association, who have advised a minimum session duration of 30 min for aerobic exercise (Colberg et al., 2016), and that exercise was performed on 12 consecutive days. Previous research has pointed out that the beneficial effects of exercise are short lived and start diminishing within 48–96 h postexercise (Kirwan et al., 2017). Therefore, it is advisable that training schedules for people with T2DM are continuous, or at least consistent, in order to sustain the advantageous effects.

It should be noted that the exercise intensity of the training sessions was adjusted dynamically to maintain a heart rate of 65% of the ${\dot{V}}_{O_{2}\mathrm{peak}}$ of each individual, which does not negate fluctuations in ${\dot{V}}_{O_{2}}$ despite heart rate consistency. However, owing to the short nature of the training intervention, there were only small changes in the ${\dot{V}}_{O_{2}\mathrm{peak}}$, and therefore, this was not expected to have a large influence.

Participants in this study had diagnosed T2DM for several years prior to participation in the study, which might make T2DM (pheno)reversion more challenging. An intervention such as the one presented in the present study might have a larger beneficial effect on preclinical individuals (e.g., overweight or obese individuals with impaired fasted glucose or impaired glucose tolerance but not T2DM).

Finally, it should be noted that most T2DM participants included in the present study were on oral hypoglycaemic medication. Participants were advised to refrain from medication intake prior to the two OGTTs; however, it cannot be confirmed that these instructions were adhered to or that there was no influence of these medications remaining in the system.

## 5 CONCLUSION

We investigated the effects of a 12 day aerobic exercise intervention on a targeted panel of T2DM‐related metabolites in fasting conditions and in response to both an OGTT and acute exercise in overweight and obese people with T2DM. We found that the fasted metabolome of T2DM participants still differed from healthy control subjects after the exercise intervention, with metabolites such as (branched‐chain) amino acids, VLDL triglycerides, cholesterol and phospholipids, apolipoprotein B100/apolipoprotein A1 ratio and glycoprotein signals GlycA and GlycB being elevated, but HDL phospholipids, SPC2 and SPC3 being decreased in people with T2DM when contrasted with healthy control subjects. The OGTT time trajectories of glycolysis‐related metabolites of T2DM participants showed abnormalities when compared with previously reported healthy responses, and the exercise intervention was not able to reverse these abnormalities. Notably, LPI(18:0) and several phosphatidylcholines had lower levels during OGTT 2 than OGTT 1, which is expected to be beneficial but requires further investigation. T2DM participants showed similar metabolic response to acute exercise at the end as at the start of the intervention, with increases in FFAs and lactate, but decreases in glucose, (branched‐chain) amino acids and (lyso)phosphatidylcholines. Future research should aim to investigate the effectiveness of exercise interventions with different durations, intensities and modalities (i.e., endurance, interval and resistance) in improving the metabolic disturbances associated with T2DM.

## AUTHOR CONTRIBUTIONS

The ^1^H NMR and LC‐MS experiments were conducted at the Australian National Phenome Centre (Murdoch University, Perth, WA, Australia). Maartje Cox analysed data, interpreted results of experiments, prepared figures, drafted the manuscript, edited the manuscript and approved the final version of manuscript. Aaron Raman conceived and designed research, performed experiments and approved the final version of manuscript. Timothy J. Fairchild conceived and designed research, interpreted results of experiments, edited and revised the manuscript and approved the final version of manuscript. John P. Beilby conceived and designed research (BHAS), edited and revised the manuscript and approved the final version of the manuscript. Bu B. Yeap conceived and designed research (BHAS), edited and revised the manuscript and approved the final version of the manuscript. Jeremy K. Nicholson interpreted data, edited and revised the manuscript and approved the final version of the manuscript. Julien Wist analysed and interpreted data, edited and revised the manuscript and approved the final version of the manuscript. Jeremiah Peiffer conceived and designed research, edited and revised the manuscript and approved the final version of the manuscript. Nathan G. Lawler performed experiments, analysed data, interpreted results of experiments, edited and revised the manuscript and approved the final version of the manuscript. All authors listed approved the final version of the manuscript and agree to be accountable for all aspects of the work in ensuring that questions related to the accuracy or integrity of any part of the work are appropriately investigated and resolved. All authors qualify for authorship, and all those who qualify for authorship are listed.

## CONFLICT OF INTEREST

None deeclared.

## Data Availability

The quantified NMR and lipidomics data are made available in the Zenodo data repository (10.5281/zenodo.15194774).

## 1

Figure A1, A2, A3, A4, A5, A6, A7, A8

Table A1, A2, A3

**TABLE A1:** Metabolites selected for analysis.

Alanine	L6TG	CER(20:0)	PC(14:0_18:2)
Creatine	L1CH	CER(20:1)	PC(14:0_18:3)
Glutamic acid	L2CH	CER(22:0)	PC(16:0_18:0)
Glutamine	L3CH	CER(22:1)	PC(16:0_18:1)
Glycine	L4CH	CER(24:0)	PC(16:0_18:2)
Histidine	L5CH	CER(24:1)	PC(16:0_18:3)
Isoleucine	L6CH	CER(26:0)	PC(16:1_18:2)
Leucine	L1FC	CER(26:1)	PC(18:0_18:0)
Lysine	L2FC	DAG(16:1_16:1)	PC(18:0_18:1)
Phenylalanine	L3FC	DAG(14:0_18:1)	PC(18:0_18:2)
Tyrosine	L4FC	DAG(16:0_18:1)	PC(18:0_18:3)
Valine	L5FC	FFA(18:0)	PC(18:1_18:1)
Lactic acid	L6FC	FFA(18:1)	PC(18:1_18:2)
3‐Hydroxybutyric acid	L1PL	FFA(18:2)	PC(18:1_18:3)
Acetoacetic acid	L2PL	FFA(18:3)	PC(18:2_18:2)
Acetone	L3PL	FFA(20:0)	PC(18:2_18:3)
Pyruvic acid	L4PL	FFA(20:1)	PC(14:0_20:2)
Glucose	L5PL	FFA(20:2)	PC(14:0_20:3)
TPTG	L6PL	FFA(20:3)	PC(14:0_20:4)
TPCH	L1AB	FFA(20:4)	PC(14:0_20:5)
LDCH	L2AB	FFA(20:5)	PC(16:0_20:1)
HDCH	L3AB	FFA(22:4)	PC(16:0_20:2)
ABA1	L4AB	FFA(22:5)	PC(16:0_20:3)
VLTG	L5AB	FFA(22:6)	PC(16:0_20:4)
IDTG	L6AB	FFA(24:0)	PC(16:0_20:5)
LDTG	H1TG	FFA(24:1)	PC(18:0_20:0)
HDTG	H2TG	FFA(14:0)	PC(18:0_20:1)
VLCH	H3TG	FFA(14:1)	PC(18:0_20:2)
VLFC	H4TG	FFA(16:0)	PC(18:0_20:3)
LDFC	H1CH	FFA(16:1)	PC(18:0_20:4)
HDFC	H2CH	FFA(16:2)	PC(18:0_20:5)
VLPL	H3CH	LPC(14:0)	PC(18:1_20:1)
IDPL	H4CH	LPC(16:0)	PC(18:1_20:2)
LDPL	H1FC	LPC(16:1)	PC(18:1_20:3)
HDPL	H2FC	LPC(18:0)	PC(18:1_20:4)
V1TG	H3FC	LPC(18:1)	PC(18:1_20:5)
V2TG	H4FC	LPC(18:2)	PC(18:2_20:1)
V3TG	H1PL	LPC(18:3)	PC(18:2_20:2)
V4TG	H2PL	LPC(20:0)	PC(18:2_20:3)
V5TG	H3PL	LPC(20:1)	PC(18:2_20:4)
V1CH	H4PL	LPC(20:2)	PC(18:2_20:5)
V2CH	H1A1	LPC(20:3)	PC(20:0_20:2)
V3CH	H2A1	LPC(20:4)	PC(20:0_20:3)
V4CH	H3A1	LPC(20:5)	PC(20:0_20:4)
V5CH	H4A1	LPC(22:4)	PC(14:0_22:4)
V1FC	H1A2	LPC(22:5)	PC(14:0_22:5)
V2FC	H2A2	LPC(22:6)	PC(14:0_22:6)
V3FC	H3A2	LPE(16:0)	PC(16:0_22:4)
V4FC	H4A2	LPE(16:1)	PC(16:0_22:5)
V5FC	SPC_All	LPE(18:0)	PC(16:0_22:6)
V1PL	SPC1	LPE(18:1)	PC(18:0_22:4)
V2PL	SPC2	LPE(18:2)	PC(18:0_22:5)
V3PL	SPC3	LPE(18:3)	PC(18:0_22:6)
V4PL	SPC3_2	LPE(20:1)	PC(18:1_22:4)
V5PL	Glyc_All	LPE(20:3)	PC(18:1_22:5)
L1TG	GlycA	LPE(20:4)	PC(18:1_22:6)
L2TG	GlycB	LPE(22:5)	PC(18:2_22:4)
L3TG	CER(14:0)	LPE(22:6)	PC(18:2_22:5)
L4TG	CER(16:0)	LPI(18:0)	PC(18:2_22:6)
L5TG	CER(18:0)	PC(14:0_18:1)	PC(20:0_22:4)
			PC(20:0_22:5)

*Note*: *where *x* is a number from 1 to 6.

Abbreviations: ABA1, apolipoprotein B100/apolipoprotein A1 ratio; CER, ceramide; DAG, diacylglycerol; FFA, free fatty acid; GlycA, glycoprotein A; Glyc_All, glycoprotein total; GlycB, glycoprotein B; HDCH, cholesterol high density lipoprotein (HDL) main fraction; HDFC, free cholesterol HDL main fraction; HDPL, phospholipid HDL main fraction; HDTG, triglyceride HDL main fraction; HxA1*, apolipoprotein A1 HDL subfraction; HxA2*, apolipoprotein A2 HDL subfraction; HxCH*, cholesterol HDL subfraction; HxFC*, free cholesterol HDL subfraction; HxPL*, phospholipid HDL subfraction; HxTG*, triglyceride HDL subfraction; IDPL, phospholipid intermediate density lipoprotein (IDL) main fraction; IDTG, triglyceride IDL main fraction; LDCH, cholesterol low density lipoprotein (LDL) main fraction; LDFC, free cholesterol LDL main fraction; LDPL, phospholipid LDL main fraction; LPC, lysophosphatidylcholine; LPE, lysophosphatidylethanolamine; LPI, lysophosphatidylinositol; LDTG, triglyceride LDL main fraction; LxAB*, apolipoprotein B LDL subfraction; LxCH*, cholesterol LDL subfraction; LxFC*, free cholesterol LDL subfraction; LxPL*, phospholipid LDL subfraction; LxTG*, triglyceride LDL subfraction; PC, phosphatidylcholine; SPC_All, supramolecular phospholipid composite total; SPCx*, supramolecular phospholipid composite x; TPCH, total cholesterol lipoproteins; TPTG, total triglycerides lipoproteins; VLCH, cholesterol very low density lipoprotein (VLDL) main fraction; VLFC, free cholesterol VLDL main fraction; VLPL, phospholipid, VLDL main fraction; VLTG, triglyceride VLDL main fraction; VxCH*, cholesterol VLDL subfraction; VxFC*, free cholesterol VLDL subfraction; VxPL*, phospholipid VLDL subfraction; VxTG*, triglyceride VLDL subfraction.

**TABLE A2:** Adjusted *p*‐values for multi‐group comparisons of demographics.

Parameter	T2DM pre‐intervention versus T2DM postintervention	T2DM pre‐intervention versus healthy control subjects	T2DM pre‐intervention versus T2DM control subjects	T2DM postintervention versus healthy control subjects	T2DM postintervention versus T2DM control subjects	Healthy controls versus T2DM control subjects
Age, years		0.037	<0.0001			<0.0001
BMI, kg/m^2^	0.931	<0.0001	1.000	<0.0001	1.000	<0.0001
Weight, kg	0.809	<0.0001	1.000	<0.0001	1.000	<0.0001
Fat, %	0.051					
VAT, g	0.450					
HbA_1c_, %		<0.0001	0.389			<0.0001
CRP	0.079	<0.0001	0.845	<0.0001	1.000	<0.0001
FBG, mmol/L	0.301					
2 h Glucose AUC, mmol/L	0.135					
${\dot{V}}_{O_{2}\mathrm{peak}}$, mL/kg/min	0.011					

Abbreviations: AUC, area under the curve; BMI, body mass index; CRP, C‐reactive protein; FBG, fasted blood glucose; HbA_1c_, haemoglobin A_1c_; T2DM, type 2 diabetes mellitus; VAT, visceral adipose tissue; ${\dot{V}}_{O_{2}\mathrm{peak}}$, peak oxygen uptake during exercise.

**TABLE A3:** Metabolite loadings on the first and second principal component corresponding to the principal component analysis scores plot in Figure 2.

Principal component 1		Principal component 2	
Metabolite	Loading	Metabolite	Loading
VLPL	0.148	Glucose	0.025
VLTG	0.145	3‐Hydroxybutyric acid	0.020
VLFC	0.144	Alanine	0.018
V1PL	0.144	Leucine	0.014
V3PL	0.143	Acetone	0.014
V1CH	0.141	Lactic acid	0.013
V1FC	0.141	Isoleucine	0.012
V3FC	0.141	Phenylalanine	0.008
V2PL	0.141	Glutamic acid	0.008
V1TG	0.140	Acetoacetic acid	0.007
VLCH	0.140	Lysine	0.001
TPTG	0.140	Valine	0.000
V2TG	0.138	H1A1	−0.001
V3TG	0.138	Pyruvic acid	−0.007
V2FC	0.135	Histidine	−0.010
V3CH	0.135	Glutamine	−0.012
IDTG	0.134	H1CH	−0.012
V4TG	0.134	H1PL	−0.014
V4PL	0.133	Tyrosine	−0.016
V2CH	0.132	H1TG	−0.017
H4TG	0.128	GlycB	−0.019
GlycA	0.128	Glycine	−0.025
Glyc_All	0.125	V1TG	−0.031
V4CH	0.124	V1PL	−0.031
IDPL	0.120	V1FC	−0.040
V4FC	0.120	H1FC	−0.041
L6TG	0.101	V5FC	−0.041
V5PL	0.098	H2TG	−0.043
L5TG	0.098	H2CH	−0.043
H3TG	0.096	SPC_Glyc	−0.043
SPC3_2	0.094	V1CH	−0.044
V5FC	0.091	VLTG	−0.046
Isoleucine	0.087	V2PL	−0.047
V5CH	0.086	HDCH	−0.048
L6AB	0.080	H4TG	−0.048
HDTG	0.078	V2TG	−0.048
ABA1	0.076	V5CH	−0.048
V5TG	0.076	H1A2	−0.048
Glucose	0.072	SPC3_2	−0.050
Glutamic acid	0.067	V5PL	−0.050
Valine	0.067	VLPL	−0.050
LDTG	0.065	HDTG	−0.051
L5AB	0.063	V2FC	−0.051
H2TG	0.062	IDTG	−0.052
L6CH	0.060	H2PL	−0.052
GlycB	0.059	V3FC	−0.054
Alanine	0.056	V3TG	−0.055
L6PL	0.055	V3PL	−0.055
L1TG	0.052	TPTG	−0.057
L5PL	0.051	Glyc_All	−0.057
L5CH	0.047	HDFC	−0.059
Leucine	0.046	V2CH	−0.060
L4TG	0.045	GlycA	−0.060
Pyruvic acid	0.044	VLFC	−0.061
Tyrosine	0.041	H3TG	−0.061
Lactic acid	0.040	H2A1	−0.062
L6FC	0.028	V5TG	−0.062
Phenylalanine	0.025	VLCH	−0.063
Acetone	0.021	HDPL	−0.065
L5FC	0.019	V4TG	−0.066
H4A2	0.017	V3CH	−0.067
SPC1	0.014	L6TG	−0.070
Lysine	0.005	H4CH	−0.071
Creatine	0.002	Creatine	−0.073
Acetoacetic acid	0.002	L2FC	−0.074
3‐Hydroxybutyric acid	0.000	L2CH	−0.074
H4A1	−0.005	SPC2	−0.076
H4PL	−0.006	V4PL	−0.077
Histidine	−0.008	L2PL	−0.078
L4AB	−0.008	H2FC	−0.080
H1TG	−0.010	H4A2	−0.085
SPC3	−0.014	L2AB	−0.086
L4PL	−0.022	V4CH	−0.089
L4CH	−0.023	H3CH	−0.089
TPCH	−0.023	H4A1	−0.090
H3A2	−0.028	H3PL	−0.094
H4CH	−0.037	H3A1	−0.094
L3TG	−0.038	V4FC	−0.095
H4FC	−0.041	L6CH	−0.099
Glutamine	−0.049	H4PL	−0.099
Glycine	−0.050	SPC1	−0.099
LDCH	−0.052	L6AB	−0.099
LDPL	−0.053	L6PL	−0.102
L4FC	−0.054	L6FC	−0.103
L2TG	−0.060	H2A2	−0.104
H2A2	−0.061	L3FC	−0.106
LDFC	−0.071	L1TG	−0.113
H3PL	−0.071	H4FC	−0.116
L1AB	−0.072	IDPL	−0.117
L1PL	−0.073	ABA1	−0.118
L1CH	−0.076	SPC_All	−0.119
H3A1	−0.078	L5TG	−0.121
L1FC	−0.082	H3FC	−0.125
H3FC	−0.086	L3CH	−0.128
H1A2	−0.086	H3A2	−0.130
SPC_All	−0.089	L3PL	−0.132
L3AB	−0.095	L2TG	−0.132
H2A1	−0.097	L3AB	−0.137
H2PL	−0.100	L5CH	−0.141
L3CH	−0.101	L5PL	−0.143
H3CH	−0.102	L1CH	−0.143
L3PL	−0.103	L5AB	−0.143
L2AB	−0.104	L1AB	−0.144
HDPL	−0.104	L1PL	−0.145
H1A1	−0.105	L1FC	−0.146
SPC2	−0.106	L4TG	−0.147
H2FC	−0.107	LDTG	−0.149
H1PL	−0.110	L5FC	−0.152
L2CH	−0.111	L3TG	−0.156
L2PL	−0.113	L4FC	−0.159
H1CH	−0.117	L4CH	−0.160
H2CH	−0.118	L4PL	−0.162
L2FC	−0.123	LDFC	−0.164
H1FC	−0.123	L4AB	−0.165
L3FC	−0.124	LDCH	−0.174
HDCH	−0.125	SPC3	−0.181
HDFC	−0.126	LDPL	−0.181
SPC_Glyc	−0.131	TPCH	−0.205

*Note*: *where *x* is a number from 1 to 6.

Abbreviations: ABA1, apolipoprotein B100/apolipoprotein A1 ratio; CER, ceramide; DAG, diacylglycerol; FFA, free fatty acid; GlycA, glycoprotein A; Glyc_All, glycoprotein total; GlycB, glycoprotein B; HDCH, cholesterol high density lipoprotein (HDL) main fraction; HDFC, free cholesterol HDL main fraction; HDPL, phospholipid HDL main fraction; HDTG, triglyceride HDL main fraction; HxA1*, apolipoprotein A1 HDL subfraction; HxA2*, apolipoprotein A2 HDL subfraction; HxCH*, cholesterol HDL subfraction; HxFC*, free cholesterol HDL subfraction; HxPL*, phospholipid HDL subfraction; HxTG*, triglyceride HDL subfraction; IDPL, phospholipid intermediate density lipoprotein (IDL) main fraction; IDTG, triglyceride IDL main fraction; LDCH, cholesterol low density lipoprotein (LDL) main fraction; LDFC, free cholesterol LDL main fraction; LDPL, phospholipid LDL main fraction; LPC, lysophosphatidylcholine; LPE, lysophosphatidylethanolamine; LPI, lysophosphatidylinositol; LDTG, triglyceride LDL main fraction; LxAB*, apolipoprotein B LDL subfraction; LxCH*, cholesterol LDL subfraction; LxFC*, free cholesterol LDL subfraction; LxPL*, phospholipid LDL subfraction; LxTG*, triglyceride LDL subfraction; PC, phosphatidylcholine; PCA, Principal Component Analysis; SPC_All, supramolecular phospholipid composite total; SPCx*, supramolecular phospholipid composite x; TPCH, total cholesterol lipoproteins; TPTG, total triglycerides lipoproteins; VLCH, cholesterol very low density lipoprotein (VLDL) main fraction; VLFC, free cholesterol VLDL main fraction; VLPL, phospholipid, VLDL main fraction; VLTG, triglyceride VLDL main fraction; VxCH*, cholesterol VLDL subfraction; VxFC*, free cholesterol VLDL subfraction; VxPL*, phospholipid VLDL subfraction; VxTG*, triglyceride VLDL subfraction.
